# Implementation of an Early Childhood Healthy Eating and Physical Activity Program in New South Wales, Australia: *Munch & Move*

**DOI:** 10.3389/fpubh.2020.00034

**Published:** 2020-02-21

**Authors:** Amanda M. Green, Seema Mihrshahi, Christine Innes-Hughes, Blythe J. O'Hara, Bronwyn McGill, Chris Rissel

**Affiliations:** ^1^NSW Office of Preventive Health, Liverpool, NSW, Australia; ^2^Sydney School of Public Health, Sydney Medical School, University of Sydney, Sydney, NSW, Australia; ^3^NHMRC Centre of Research Excellence in the Early Prevention of Obesity in Childhood, Sydney School of Public Health, University of Sydney, Sydney, NSW, Australia; ^4^Charles Perkins Centre, University of Sydney, Sydney, NSW, Australia; ^5^Prevention Research Collaboration, Sydney School of Public Health, University of Sydney, Sydney, NSW, Australia

**Keywords:** childhood obesity, early childhood, health promotion, healthy eating, physical activity

## Abstract

**Background:** Childhood obesity is an important public health issue. Approximately 20% of 2–4 year olds are overweight or obese, meaning 1 in 5 Australian children start school above a healthy weight. In the state of New South Wales (NSW) the combined prevalence of childhood overweight and obesity is significantly higher among children from low socioeconomic status backgrounds and children from regional, rural and remote areas. This paper describes implementation of a healthy eating and active play program (*Munch & Move*) for center-based early childhood education and care (ECEC) services aimed at influencing healthy behaviors in young children in NSW, Australia. It shows changes over time including a focus on disadvantaged, Aboriginal and remote communities. It also discusses the challenges and future opportunities for the program.

**Methods:** Routine data in relation to service delivery (reach) and implementation indicators are collected by Local Health District staff. Fifteen implementation indicators (known as practices) were introduced to monitor the implementation of *Munch & Move* (six related to promoting and encouraging healthy eating, four related to improving physical activity, two related to small screen recreation; and three related to quality of service delivery).

**Results:** As of 30 June 2017, 88.4% of ECEC services have staff trained in *Munch & Move*. Of the 15 practices related to promoting and encouraging healthy eating, increasing physical activity and improving the quality of service delivery 13 practices saw significant improvements between 2012 and 2017. This was consistent for services with a high proportion of Aboriginal children and for services in disadvantaged and remote communities. There has been a statistically significant increase in the proportion (37.6–81.0%, *p* < 0.0001) and type of ECEC services (preschools 36.1–81.3%, *p* < 0.0001, long day care 38.6–81.0%, *p* < 0.0001, and occasional care 34.0–74.6%, *p* < 0.0001) that have implemented the program since 2012 as well as in services with a high proportion of Aboriginal children (33.6–85.2% *p* < 0.0001), services in disadvantaged communities (37.4–83.3% *p* < 0.001), and services in remote communities (27.8–59.4% *p* < 0.0139).

**Discussion:** This paper demonstrates that *Munch & Move* has seen large improvements in the delivery of training, practice achievements and program adoption in ECEC services across NSW including services in disadvantaged and remote communities and that have a higher proportion of Aboriginal children.

## Background

Childhood obesity is one of the most important public health issues facing Australia. Approximately 20% of 2–4 year olds are overweight or obese (1), meaning 1 in 5 Australian children start school above a healthy weight (2). A study conducted in New South Wales (NSW) found the combined prevalence of overweight and obesity among primary school children was significantly higher among children from low socioeconomic status (SES) backgrounds (35%) than children from high SES backgrounds (18.9%) and the prevalence significantly increased among children from regional, rural, and remote areas from 17.4% in 2010 to 21.1% in 2015 (2). Children from Aboriginal and Torres Strait Islander (Indigenous) communities are also more likely to be overweight or obese than non-Indigenous children (30% compared with 25%) (3).

Early childhood is an important period to establish healthy eating and physical activity behaviors (4, 5), and provides a critical time to implement obesity prevention initiatives. Children spend a significant amount of time in early childhood settings including preschools, long day care and family day care services (6). These services present an opportunistic setting to promote and establish good healthy eating and physical activity habits. They also provide a setting where the organizational infrastructure required for health promotion activities is available and responsive to program implementation. The *Get Up & Grow* guidelines in Australia (7) and the *Early Childhood Obesity Prevention Policies* in United States (8) are specific guidelines and recommendations which have been developed to promote healthy environments for young children and prevent obesity in early childhood. The World Health Organization (WHO) strongly recommends that guidance on, and support for key obesity prevention behaviors in early childhood be provided to ensure children grow appropriately and develop healthy habits (9). The WHO implementation plan recommends that settings like child care create healthy food environments and incorporate physical activity into daily routines (10). The plan outlines key elements for implementation of programs including capacity building, advocacy, expanding the knowledge base, mobilizing resources and most importantly, monitoring and accountability.

The *Munch & Move* program is a key state-wide healthy eating and active play program aimed at influencing the healthy eating and physical activity environments of young children aged from birth to 5 years. *Munch & Move* is designed to build capacity in center-based early childhood education and care (ECEC) services (preschools, long day care, and occasional care) to promote and encourage children's healthy eating and physical activity and reduce small screen recreation in young children, as well as influence policies and practices in the service. The program aligns with the key elements of the WHO implementation plan (10).

Evidence shows that regulation and educational workshops and training for child care providers on nutrition, physical activity and screen-time behaviors increased knowledge, improved center policies and reduced body mass index for children in child care centers in the United States (11, 12). In Australia, obesity prevention interventions in preschools have shown significant positive changes in children's food intake, movement skills, indicators of weight status, and reduction in the prevalence of overweight and obesity (13–16). A written physical activity policy, structured staff-led physical activity and staff participating in active play have also been associated with higher levels of physical activity in preschools (16). It has also been demonstrated through initial implementation of Munch and Move in pre-schools that it is a feasible, acceptable, and appropriate way to build knowledge and skills of early childhood professionals (17).

There are two aims of this paper. The first is to describe training and implementation of the *Munch & Move* program over time with a focus on implementation in disadvantaged, Aboriginal and remote communities. The second aim is to discuss the challenges and future opportunities for the program.

## Methods

### Description of the *Munch & Move* Program

*Munch & Move* was launched in July 2008 initially targeting children from 3 to 5 years of age attending preschools in NSW. In 2010 the program was scaled-up to be state-wide and inclusive of long day care and occasional care services covering children aged from birth to 5 years (18). More information on the *Munch & Move* program is provided on the NSW Healthy Kids website (www.healthykids.nsw.gov.au/campaigns-programs/about-munch-move.aspx). To create an environment that is supportive of healthy eating and physical activity *Munch & Move* is based on six health promoting key messages–encourage and support breastfeeding, choose water as a drink, choose healthier snacks, eat more fruit and vegetables, get active each day and turn off the screen and get active.

*Munch & Move* offers professional development training for early childhood educators to implement a fun, play-based approach to supporting healthy eating and physical activity habits in young children. It also offers practical resources (i.e., resource manual, poster, instructional videos for fundamental movement skills and a music CD) to support service policies and practices, support visits and regular contact from Local Health District (LHD) health professionals and fact sheets to communicate the key messages with families (18). The program is implemented using a “whole-of-service” health promotion approach (10, 19) which means bringing together directors, management, educators, staff, children, and families to create an environment that is supportive of healthy eating and physical activity. The program is strongly aligned to the National Quality Framework (NQF) (7) and in particular to children's health and safety, which outlines specific standards relating to the provision of healthy food and drinks, and the promotion of physical activity through planned and spontaneous experiences (20, 21).

Program training is delivered to early childhood educators working in ECEC services by an early childhood registered training organization. From July 2008 to April 2015 362 full day face-to-face workshops were delivered and from July 2015 to June 2017 training was delivered via 51 “live” webinar series consisting of four 2 h modules as this was seen to be more flexible and accessible for educators. The objective of the training is to provide early childhood educators with information on the *Munch & Move* key messages, the *Australian Dietary Guidelines* and *Physical Activity and Sedentary Behavior Recommendations*, how to engage children in healthy eating related learning experiences, how to increase the amount of physical activity children engage in and how to develop their fundamental movement skills. Ongoing LHD support allows the program to be tailored to the needs of the local community and each individual service for example local workshops on healthy eating experiences and resources for Aboriginal families.

### Description of the Early Childhood Education and Care Context

As of September 2017 there were 3,753 approved ECEC services (excluding family day care and outside school hours care) in NSW (22) which is a significant increase from 2012 where there were around 2,600 (23). Of these there were 38 (1%) services located in remote locations and 1,945 (51.8%) services in low SES areas (24). In NSW there were ~596,234 children aged 0–5 years (25) and ~390,790 attended an ECEC service (6).

### Evaluation and Monitoring Framework

The monitoring framework on which this paper is based is described in Farrell et al. (26) It includes routine data collected by LHDs in relation to service delivery indicators (i.e., number and classification of ECEC services across NSW and number trained in *Munch & Move*). It also includes implementation indicators (i.e., number of practices achieved and not achieved by services).

Because the prevalence of childhood obesity is higher in disadvantaged, Aboriginal and remote communities, the implementation of *Munch & Move* is also monitored and reported for these three types of communities. Disadvantaged communities are those communities located in the two most disadvantaged communities as measured by the Socio-economic Index for Areas [SEIFA quintiles 1 and 2] (27). Aboriginal communities are those communities with a high proportion of Aboriginal children in their care (10% or more children identified as being from Aboriginal background). Communities are classified as being remote based on their postcode (defined by the Accessibility/Remoteness Index of Australia [ARIA] classifications) (28).

Dodds et al. conducted a validation study of certain measures to implement and monitor obesity prevention policies and practices (29). Fifteen program adoption indicators (known as practices) were modified from this validated instrument and introduced to monitor the implementation of *Munch & Move* across NSW and to inform LHD service delivery. The monitoring framework for center-based ECEC services includes four domains and 15 practices, as detailed in Table 1.

**Table 1:** Program adoption indicators (practices).

Encouraging healthy eating	1. Lunchboxes monitored daily
	2. Fruit and vegetables on menu
	3. Only healthy snacks on the menu
	4. Water or age-appropriate drinks
	5. Healthy eating learning experiences at least 2 times per week
Daily physical activity	6. Tummy time for babies
	7. Active playtime for at least 25% of opening hours (ages 1–5 years)
	8. Fundamental movement skills (ages 3–5 years)
	9. Appropriate small-screen recreation (ages 3–5 years)
Policies	10. Written nutrition policy
	11. Written physical activity policy
	12. Written policy restricting small screen recreation
Educating & monitoring	13. Provide health information to families
	14.50% of educators trained in nutrition and 50% in physical activity
	15. Monitoring and reporting annually

To ensure consistent data collection by LHDs a monitoring guide was developed. It includes detailed practices and questions to measure achievement (see Table S1).

### Data Sources and Analysis

A list of approved ECEC services across NSW was obtained from a government regulatory agency and is updated by LHDs on a regular basis. This list includes service name and license code, service type (i.e., preschool, long day care), local government area, contact details and opening days and hours. Practice data was initially collected via a computer assisted telephone interview (CATI) through self-reporting by a service representative. Subsequent data has been collected by trained health promotion officers through regular scheduled support visits, where data were collected through observation and conversations with the service representative.

Data on practices were collected using the monitoring guide (see Table S1). Data are stored in the NSW Health Population Health Information Management System (PHIMS). This system enables monitoring and reporting of the implementation of Healthy Children Initiative programs (30).

From July 2012 to June 2016 program adoption was measured and reported with reference to the number of services achieving 70% (or more) of the practices that were relevant for their particular service. From July 2016 to June 2017 the benchmark was increased to drive continuous quality improvement of program implementation. The new benchmark was the number of services achieving 80% (or more) of the practices that are relevant for their particular service.

#### Data Analysis

Data were reported as percentages (%) or proportion of the total of ECEC services achieving training, practice achievement, and program adoption targets. Chi-squared tests for differences in proportion were used to ascertain differences between data collection points as well as difference of training, practice and adoption measures between type of service and priority groups. Data analysis was performed using Microsoft Excel 2013. Differences were regarded as statistically significant at the *p* < 0.05 level.

## Results

Data relating to training has been collected since July 2008 and monitoring of program implementation has been collected since July 2012.

### Training

Trained is defined as attendance at a workshop or completion of a webinar series. As of 30 June 2017, 88.4% (*n* = 3,328) of center-based ECEC services had staff trained in *Munch & Move* (e.g., at least one staff had attended a workshop or completed a webinar series). There were variations between the type of services that had taken part in the training, with a significantly higher proportion of preschools having their staff trained (*n* = 830, 92.0%) compared to long day care (*n* = 2,424, 87.6%, chi-squared = 11.959, *p* = 0.0005) and other center-based services (*n* = 17, 56.7%, chi-squared = 26.080, *p* < 0.0001), which included distance learning, early intervention, and mobile services. For occasional care services 90.5% (*n* = 17) of staff were trained and this was not significantly different from preschool (chi-squared = 0.051, *p* = 0.8219 or long day care services (chi-squared = 0.131, *p* = 0.7176).

The proportion of ECEC services whose staff had undergone training was significantly higher for services with a higher proportion of Aboriginal children (*n* = 255, 95.3%, chi-squared = 11.416, *p* = 0.0007) and for services in disadvantaged communities (*n* = 1369, 92.8%, chi-squared = 21.506, *p* < 0.0001) when compared to the NSW average. Services in remote communities also had higher training attendance (*n* = 34, 94.1%) when compared to the NSW average, however, this was not significantly different (chi-squared = 1.071, *p* = 0.3008).

### Practice Achievement Over Time

Practice achievement is defined as “met” in accordance with the monitoring guide. There have been substantial increases in practice achievements made by ECEC services since 2012 (Table 2). Of the 15 practices there were significant improvements in 13 practices between 2012 and 2017. There were only two practices where no significant differences occurred and these were having a written nutrition policy (already at 98%) and having 50% of primary contact educators trained in nutrition and physical activity. Two practices that already had relatively high levels of achievement in 2012 but decreased in 2017 were related to small screen recreation, namely appropriate use of small screen recreation and a written policy restricting small screen recreation.

**Table 2:** Practice achievement over time.

	**2012**		**2017**		Chi-squared value^*^	***p***
**Practice**	***n***	**%**	***n***	**%**		
1. Lunchboxes monitored daily	291	84.6	1,430	96.9	82.2210	0.0001
2. Fruit and vegetables at least once per day	249	90.2	1,857	96.3	21.0590	0.0001
3. Only healthy snacks on the menu	209	75.7	1,968	92.5	80.7120	0.0001
4. Water or age appropriate drinks every day	323	52.4	2,744	82.1	263.2120	0.0001
5. Healthy eating learning experiences at least 2 times per week	418	67.8	3,071	91.8	285.3150	0.0001
6. Tummy time for babies every day	301	89.1	1,806	96.4	33.9470	0.0001
7. Physical activity for at least 25% of the daily opening hours (ages 1–5 years)	556	90.1	3,206	95.9	36.8570	0.0001
8. Daily fundamental movement skills (ages 3–5 years)	395	64.3	2,398	72.1	15.2770	0.0001
9. Appropriate use of small screen recreation (ages 3–5 years)	590	95.6	3,115	93.6	3.6410	0.0564
10. Written nutrition policy	606	98.2	3,286	98.3	0.0310	0.8605
11. Written physical activity policy	360	58.4	2,733	81.7	165.1580	0.0001
12. Written policy restricting small screen recreation	528	85.6	2,740	81.9	4.9330	0.0264
13. Provide health information to families within past 12 months	248	40.2	2,684	80.3	435.8160	0.0001
14.50% of primary contact educators trained in nutrition and 50% trained in physical activity	308	49.9	1,852	55.4	6.3530	0.0117
15. Monitoring and reporting annually	392	63.5	3,092	92.5	414.2180	0.0001

*Data source: 2012 collected using a CATI and 2017 collected using PHIMS*.

* *Chi-squared values for differences in proportion between 2012 and 2017*.

### Practice Achievement Across Service Type

Practice achievements across type of service were fairly consistent, with some notable exceptions (Table 3). When compared to “All Services,” long day care services were significantly less likely to provide water or age appropriate drinks and to have 50% of contact educators trained in nutrition and physical activity. Long day care services were significantly more likely than “All Services” to have a written physical activity policy. Preschools were significantly more likely than “All Services” to provide water or age appropriate drinks every day, to provide daily fundamental movement skills and to have 50% of primary contact educators trained in nutrition and physical activity. However, they were significantly less likely than “All Services” to provide daily fruit and vegetables, only have healthy snacks, provide daily tummy time for babies and to have written policies for physical activity and restricting small screen recreation. Occasional care services were significantly more likely than “All Services” to provide water or age appropriate drinks every day, but significantly less likely to provide fruit and vegetables at least once a day, provide health information to families and undertake annual monitoring and reporting.

**Table 3:** Practice achievement by service type (2017).

**Practice**	**All Services**		**Long day care**		Chi-squared value^*^	***p***	**Preschool**		Chi-squared value^*^	***p***	**Occasional care**		Chi-squared value^*^	***p***
	***n***	**%**	***n***	**%**			***n***	**%**			***n***	**%**		
1. Lunchboxes monitored daily	1,430	96.9	633	97.2	0.139	0.709	742	96.6	0.147	0.7018	52	96.3	0.062	0.8034
2. Fruit and vegetables at least once per day	1,857	96.3	1,781	96.7	0.446	0.5044	73	88.0	14.126	0.0002	3	75.0	5.023	0.0250
3. Only healthy snacks on the menu	1,968	92.5	1,864	92.8	0.136	0.7119	98	86.7	5.021	0.0250	6	85.7	0.464	0.4960
4. Water or age appropriate drinks every day	2,744	82.1	1,915	78.2	13.667	0.0002	772	92.3	52.13	0.0001	54	94.7	6.109	0.0134
5. Healthy eating learning experiences at least 2 times per week	3,071	91.8	2,247	91.8	0	1	772	92.3	0.225	0.6356	49	86.0	2.477	0.1155
6. Tummy time for babies every day	1,806	96.4	1,682	97.0	1.012	0.3143	81	88.0	16.131	0.0001	43	93.5	1.068	0.3015
7. Physical activity for at least 25% of the daily opening hours (ages 1–5 years)	3,206	95.9	2340	95.6	0.314	0.5751	810	96.9	1.784	0.1817	53	93.0	1.185	0.2763
8. Daily fundamental movement skills (ages 3–5 years)	2,398	72.1	1,721	70.7	1.352	0.245	636	76.2	5.684	0.0171	38	66.7	0.811	0.3680
9. Appropriate use of small screen recreation (ages 3–5 years)	3,115	93.6	2,281	93.8	0.095	0.7579	780	93.4	0.044	0.8333	51	89.5	1.556	0.2122
10. Written nutrition policy	3,286	98.3	2,408	98.4	0.087	0.7684	819	98.0	0.3480	0.5552	56	98.3	0	1
11. Written physical activity policy	2,733	81.7	2,053	83.9	4.765	0.0290	637	76.2	12.935	0.0003	41	71.9	3.574	0.0587
12. Written policy restricting small screen recreation	2,740	81.9	2,050	83.7	3.194	0.0739	645	77.2	9.5820	0.0020	43	75.4	1.589	0.2074
13. Provide health information to families within past 12 months	2,684	80.3	1,956	79.9	10.943	0.0009	687	82.2	1.5490	0.2133	38	66.7	6.494	0.0108
14.50% of primary contact educators trained in nutrition and 50% trained in physical activity	1,852	55.4	1,262	51.6	8.209	0.0042	553	66.2	31.926	0.0001	35	61.4	0.817	0.3661
15. Monitoring and reporting annually	3,092	92.5	2,274	92.9	0.333	0.5640	769	92.0	0.2380	0.6256	46	80.7	10.980	0.0009

*Data source: 2017 collected using PHIMS*.

* *Chi-squared values show comparisons in proportion with all services*.

### Practice Achievement by Priority Population Group

Practice achievements across ECEC services with a high proportion of Aboriginal children, services in disadvantaged communities, and services in remote communities had some significant differences when compared with “All Services” (Table 4). Services with a high proportion of Aboriginal children were significantly more likely than “All Services” to provide water or age appropriate drinks daily, provide health information to families and to have 50% of primary contact educators trained in nutrition and physical activity, However, these services were significantly less likely than “All Services” to have appropriate use of small screen recreation. Services in disadvantaged communities were significantly more likely than “All Services” to provide water or age appropriate drinks daily and to have 50% of primary contact educators trained in nutrition and physical activity, but significantly less likely to offer only healthy snacks and have appropriate use of small screen recreation. Services in remote communities were significantly less likely than “All Services” to offer fruit and vegetables daily, have healthy eating learning experiences at least twice a week, provide daily tummy time for babies and have appropriate use of small screen recreation.

**Table 4:** Practice achievement by priority population group (2017).

**Practice**	**All services**		**Services with a high proportion of Aboriginal children attending^a^**		Chi-squared value^*^	***p***	**Services in disadvantaged communities^b^**		Chi-squared value^*^	***p***	**Services in remote communities^c^**		Chi-squared value^*^	***p***
	***n***	**%**	***n***	**%**			***n***	**%**			***n***	**%**		
1. Lunchboxes monitored daily	1,430	96.9	181	97.3	0.089	0.7652	728	96.9	0	1	23	95.8	0.095	0.7585
2. Fruit and vegetables at least once per day	1,857	96.3	66	94.3	0.744	0.3883	608	95.5	0.818	0.3659	8	80.0	7.256	0.0071
3. Only healthy snacks on the menu	1,968	92.5	85	93.4	0.102	0.7491	650	89.3	7.283	0.0070	15	83.3	2.156	0.1420
4. Water or age appropriate drinks every day	2,744	82.1	221	88.4	6.404	0.0114	1,163	85.1	6.176	0.0129	25	80.6	0.047	0.8284
5. Healthy eating learning experiences at least 2 times per week	3,071	91.8	229	91.6	0.012	0.9116	1,250	91.5	0.115	0.7347	22	78.6	6.348	0.0117
6. Tummy time for babies every day	1,806	96.4	92	96.8	0.042	0.8376	654	95.3	1.622	0.2028	9	81.8	6.564	0.0104
7. Physical activity for at least 25% of the daily opening hours (ages 1–5 years)	3,206	95.9	239	95.6	0.053	0.8180	1,306	95.6	0.218	0.6409	27	96.4	0.018	0.8943
8. Daily fundamental movement skills (ages 3–5 years)	2,398	72.1	181	72.4	0.010	0.9188	954	70.0	2.091	0.1482	16	57.1	3.096	0.0785
9. Appropriate use of small screen recreation (ages 3–5 years)	3,115	93.6	217	86.8	16.789	0.0001	1,240	91.0	9.833	0.0017	20	71.4	22.239	0.0001
10. Written nutrition policy	3,286	98.3	247	98.8	0.355	0.5513	1,342	98.2	0.057	0.8112	27	96.4	0.594	0.4408
11. Written physical activity policy	2,733	81.7	204	81.6	0.002	0.9685	1,135	83.1	1.294	0.2554	22	78.6	0.178	0.6729
12. Written policy restricting small screen recreation	2,740	81.9	194	77.6	2.864	0.0906	1,118	81.8	0.007	0.9356	24	85.7	0.271	0.6028
13. Provide health information to families within past 12 months	2,684	80.3	218	87.2	7.131	0.0076	1,121	82.1	2.027	0.1545	19	67.9	3.091	0.0787
14.50% of primary contact educators trained in nutrition and 50% trained in physical activity	1,852	55.4	167	66.8	12.277	0.0005	833	61.0	12.408	0.0004	15	53.6	0.036	0.8487
15. Monitoring and reporting annually	3,092	92.5	229	91.6	0.269	0.6037	1,272	93.1	0.269	0.6037	25	89.3	0.408	0.5228

a *High proportion of Aboriginal children attending is defined as having a proportion of 10% or more identified as being from Aboriginal background*.

a *Disadvantaged communities defined as the two most disadvantaged communities as measured by the Socio-economic Index for Areas (27)*.

a *Remote communities defined as remote and very remote communities as measured by Remoteness classifications (28)*.

* *Chi-squared values show comparisons in proportion with all services*.

### Program Adoption

Program adoption is reported with reference to the number of services achieving a defined per cent of the practices that are relevant for their particular service. There has been a statistically significant increase in the proportion and type of ECEC services that have implemented *Munch & Move* since state-wide monitoring was introduced in 2012. In 2012 the total services across NSW achieving 70% (or more) of the practices was 37.6% and by June 2016 this increased to 81.0% (chi-squared = 1356.513, *p* < 0.0001). For preschools it increased from 36.1 to 81.3% (chi-squared = 366.865, *p* < 0.0001), for long day care it increased from 38.6 to 81.0% (chi-squared = 947.856, *p* < 0.0001), and for occasional care it increased from 34.0 to 74.6% (chi-squared = 19.095, *p* < 0.0001).

As of 30 June 2017, 71.6% (2,687/3,601) of ECEC services had achieved 80% (or more) of the program practices. Due to the proportion of practices to be achieved increasing from 70 to 80%, the proportion of services adopting the program between 2016 (81.0%) and 2017 (71.6%) decreased slightly; corresponding decreases in program adoption were seen for preschools (77.7%), long day care (69.8%), and occasional care services (66.7%).

There has been a statistically significant increase in the proportion of ECEC services within priority population groups that have implemented *Munch & Move*. From 2012 to 2016 services with a high proportion of Aboriginal children increased from 33.6 to 85.2% (chi-squared = 136.947, *p* < 0.0001), services in disadvantaged communities increased from 37.4 to 83.3% (chi-squared = 628.551, *p* < 0.0001) and services in remote communities increased from 27.8 to 59.4% (chi- squared = 6.053, *p* = 0.0139).

From 2016 to 2017 there was a decrease for ECEC services with a high proportion of Aboriginal children (81.2%) and disadvantaged communities (76.4%). This decrease was because of the change in the benchmark. However, program adoption for services in remote communities increased to 66.7% despite the change in the benchmark.

## Discussion

This paper has described the training and implementation of the *Munch & Move* program over time with a focus on implementation in disadvantaged, Aboriginal and remote communities. The *Munch & Move* program has influenced the healthy eating and physical activity environments in NSW ECEC services. Our findings are consistent with the Nutrition and Physical Activity Self Assessment for Child Care intervention conducted in the USA and the Romp & Chomp intervention conducted in Victoria, Australia. They support the notion that early interventions targeting ECEC services (i.e., educator knowledge and support to change service policies and practices) can create opportunities to improve children's health and well-being (12, 31).

Based on the literature, multi-component, multi-level early childhood education interventions with parental engagement are most likely to be effective for obesity prevention (32). Therefore, sustained implementation of *Munch & Move* in ECEC services is likely to be effective in supporting children to establish healthy eating and physical activity behaviors and contribute to preventing overweight and obesity. A recent analysis has shown that while there has been no increase in the prevalence of overweight and obesity in all primary school children in NSW, among 5–6 year olds this prevalence decreased from 23.9% in 2010 to 17.5% in 2015 (2). In addition, the prevalence of overweight and obesity in kindergarten children in regional, rural, and remote areas decreased from 17.4% in 2010 to 15.5% in 2015 and for low SES it decreased from 29.1% in 2010 to 27.8% in 2015.

Attendance at training across NSW is high, and is significantly higher in preschools than in long day care and occasional care services. This may be due to the program initially being available only for preschools who have therefore had longer to access training. Training is also high among priority population groups which may be a reflection of local prioritization of program delivery to these groups and access to a free program in varying training modes (i.e., workshops and webinar series).

Increases in program implementation, measured by practice achievement, have also occurred between 2012 and 2017. Some practices such as having a nutrition policy were achieved early on as they had been part of the regulations prior to the introduction of the NQF. The practice for staff on-training did not change significantly and this could be due to high staff turnover in ECEC services. The two practices that decreased during this period were related to screen time and this may be due to the increased availability and use of screens in services for educational purposes. Practice achievement across the different service types was similar and the differences may be reflective of the organizational structures (i.e., food and drinks provided by the service or by family in lunchboxes), qualifications of staff working in these service types (i.e., all educators working in preschools must have teaching qualifications so training of 50% of staff in nutrition and physical activity is not seen as necessary) and the regulation framework (i.e., occasional care services do not come under the NQF so may not have documented reporting). Practice achievement among priority population groups was significantly higher except for the appropriate use of small screen devices. ECEC services in remote communities were also less likely to offer fruit and vegetables daily, to offer only healthy snacks and to have healthy eating learning experiences at least twice a week.

This analysis reports that the reach and program adoption by ECEC services in disadvantaged and remote communities and those services with a higher proportion of Aboriginal children was similar. This demonstrates equitable program implementation, particularly for the most vulnerable communities. These results are consistent with a study conducted by Yoong et al. (33) that found adoption of best-practice healthy eating and physical activity practices did not vary by locality and SES.

### Challenges and Future Opportunities for the Program

The second aim of this paper was to discuss the challenges and future opportunities for the program. *Munch & Move* has high universal reach and program adoption and this may be due to a number of effective key intervention components outlined in the Romp & Chomp intervention evaluation and the WHO implementation plan (10, 31). These components include capacity building of early childhood educators through professional development, training, and resources accompanied by ongoing LHD support leading to organizational change over time; the introduction of the NQF in 2012 which provides a policy imperative for services and parent education materials (31).

A strength of the *Munch & Move* program is that once an ECEC service is trained they are supported by a health promotion officer from their LHD through visits, local workshops and newsletters for both the service and their families. This allows flexibility in the ongoing support provided to meet the service and community needs. Also, the monitoring guide used to collect the data is based on a modified validated tool (29).

However, there are some limitations of the data. This evaluation of *Munch & Move* reflects the “real world,” not a research trial and uses routine monitoring data which relies on self-reported data from ECEC services rather than direct observations which may contain reporter bias. Further, the data used for this paper includes a combination of two data sources (CATI and PHIMS) and inherent within this process is the limitation of comparing data from different sources.

Consistent with the WHO implementation plan (10) the NSW government recognizes the importance of obesity prevention and this is evident through the delivery of NSW Government's Premier Priorities Childhood Overweight and Obesity Delivery Plan (34) and the *NSW Healthy Eating and Active Living Strategy: Preventing overweight and obesity in New South Wales 2013–2018* (35). The *Munch & Move* program is one of the key initiatives that focuses on early childhood. In order to achieve the Premier's target, *Munch & Move* will continue to provide training and support to early childhood educators and their services across NSW including the reengagement of family day care services along with monitoring of program implementation through strengthened practices for center-based ECEC services.

In conclusion, the *Munch & Move* has seen large improvements in the delivery of training, practice achievements and program adoption in ECEC services across NSW including services in disadvantaged and remote communities and that have a higher proportion of Aboriginal children. These improvements create important opportunities to improve children's health and well-being, and should contribute to the prevention of childhood overweight and obesity. Future research could consider assessing the direct impact of programs such as *Munch & Move* on individual child health and well-being outcomes.

## Ethics Statement

The consent procedures were approved by the University of Sydney Ethics Project Number 2018/205.

## Author Contributions

AG and CI-H designed the monitoring framework of *Munch & Move*. AG and SM prepared the draft manuscript. SM, BO'H, and BM performed the data analysis. All authors provided feedback on drafts and approved the final manuscript.

### Conflict of Interest

The authors declare that the research was conducted in the absence of any commercial or financial relationships that could be construed as a potential conflict of interest.

## Abbreviations

SES: socioeconomic status
NSW: New South Wales
ECEC: early childhood education and care
LHD: Local Health District
NQF: National Quality Framework
CATI: Computer Assisted Telephone Interview
PHIMS: Population Health Information Management System.
