# Supplementary material for: Implementation of an Early Childhood Healthy Eating and Physical Activity Program in New South Wales, Australia: Munch & Move
Source: Front Public Health. 2020 Feb 21;8:34. doi: 10.3389/fpubh.2020.00034 (PMC7047441; doi:10.3389/fpubh.2020.00034)
Supplement: Supplementary file 1 [file Table_1.DOCX]

Supplementary Material

**Achieving equity in the implementation of an early childhood healthy eating and physical activity program in New South Wales, Australia: *Munch & Move***

A M Green*, S Mihrshahi, C Innes-Hughes, B J O’Hara, B McGill, C Rissel

Supplementary Table

**Table S1: Practice Monitoring Guide**

| **PRACTICE** | **MEASURE** |
| --- | --- |
| **1: Site monitors food and drinks that are in children’s lunchboxes each day** | Q - Frequency that educators monitor the lunchboxes of all children:  A - Every day that site is open |
| **2: Site menu includes fruit and vegetables at least once per day** | Q - Frequency fruit is supplied (fresh, frozen or canned, in natural juice):  A - 1 time per day  A - 2 or more times per day  A - Fruit is supplied by parent  Q - Frequency vegetables are supplied (fresh, frozen or canned):  A - 1 time per day  A - 2 or more times per day  A - Vegetables are supplied by parent |
| **3: Site menu includes only healthy snack options every day** | Q - Select the snacks supplied by the site on a regular basis:  A - Fruit or vegetable pieces or platters  A - Fruit bread (e.g. raisin toast), English or fruit muffins or pikelets  A - Wholegrain or rice crackers or rice cakes  A - Unsalted pretzels, plain popcorn (no added fat), oven baked chips (not oiled)  A - Dairy snacks (such as yoghurt, cheese, custard) |
| **4: Site supplies age appropriate drinks every day** | Q - Select the types of drinks usually supplied by the site:  A - Water  A - Plain milk  Q - For children 2 years of age and older, select type(s) of milk supplied:  A - Reduced fat |
| **5: Site provides structured and specific learning experiences about healthy eating at least 2 times per week** | Q - How often are structured and specific learning experiences about healthy eating implemented in the site’s program (e.g. experiential activities about food, cooking skills, stories, and vegetable gardens):  A - 2 - 4 times per week  A - Every day of opening |
| **6: Site provides tummy time for babies 0-12 months of age every day** | Q - Number of days babies (birth to less than 12 months of age) are usually provided with supervised floor based ‘tummy’ play time:  A - Every day of opening |
| **7: Site provides physical activity for 1-5 year olds at least 25% of the daily opening hours** | Q - Average minutes each day children 1-5 years old spend in educator-led structured active play, i.e. circle time, music, dancing or planned activities to develop movement skills, e.g. 1.5 hours = 90 minutes.  Q - Average minutes each day children 1-5 years old spend in child-initiated free physically active play, e.g. 1.5 hours = 90 minutes. |
| **8: Site provides fundamental movement skills for children 3-5 years of age every day to at least 90% of children** | Q - Average number of days per week educators lead structured activity to develop Fundamental Movement Skills (FMS) for children aged 3-5 years. E.g. During a transition activity, group or circle time or during outdoor play:  A - Every day of opening  Q - Estimate the % of 3-5 year olds that usually participate (or are encouraged to participate if special needs) in structured activities to develop FMS. |
| **9: Site use of small screen recreation by 3-5 year olds is appropriate** | Q - Select the purpose(s) children aged 3-5 years spend time watching small screen devices (e.g. TV, videos or DVDs)? Please select all that apply:  A - Not applicable (e.g. No TV/DVD)  A - To gain knowledge or share information about a specific learning area or child’s interest  A - To facilitate exploration of activity, dance or movement |
| **10: Site has a written nutrition policy** | Q - Is there a written Nutrition policy (may be combined with other policies)?  A - Yes |
| **11: Site has a written physical activity policy** | Q - Is there a written Physical Activity policy (may be combined with other policies)?  A - Yes |
| **12: Site has a written policy restricting small screen recreation** | Q - Does the site have a written policy restricting child viewing of small screen devices, e.g. TV, DVDs or Videos (may be combined with other policies)?  A - Site has no TV  A - Yes |
| **13: Site provides health information to families within past 12 months** | Q - Select the topics you have sent home to families from a recognised health authority in the last 12 months. This includes material handed directly to parents, mailed or emailed or placed in their child’s pigeon hole or bag, or information included in newsletters or at orientation: (*please select all that apply*)  A - Healthy eating for children (includes list of recommended foods for lunchboxes and lunchbox ideas)  A - Physical activity for children  A - Limiting screen time for children  A - Breastfeeding (CHILDREN AGED 0-12 MONTHS ONLY) |
| **14: Site with at least 50% of Primary Contact Educators trained in nutrition and at least 50% of their staff trained in physical activity** | Q - Number of Primary Contact Educators at this site.  Q - Number of Primary Contact Educators who have received training on child nutrition or healthy eating.  Q - Number of Primary Contact Educators who have received training on physical activity. |
| **15: Site monitors and reports achievements of healthy eating and physical activity objectives annually** | Q - Does the site monitor and report annually, internally or externally, on its achievement of the healthy eating and physical activity objectives as stated in written policies, guidelines, or other documents:  A - Yes |

** Q means question and A means answer.*
